# Challenges in the Care of Clients with Established Cardiovascular Disease: Lessons Learned from Australian Community Pharmacists

**DOI:** 10.1371/journal.pone.0113337

**Published:** 2014-11-19

**Authors:** Hanni P. Puspitasari, Parisa Aslani, Ines Krass

**Affiliations:** 1 Faculty of Pharmacy, University of Sydney, Sydney, Australia; 2 Fakultas Farmasi, Universitas Airlangga, Surabaya, Indonesia; University of Florida, United States of America

## Abstract

**Background:**

As primary healthcare professionals, community pharmacists have both opportunity and potential to contribute to the prevention and progression of chronic diseases. Using cardiovascular disease (CVD) as a case study, we explored factors that influence community pharmacists’ everyday practice in this area. We also propose a model to best illustrate relationships between influencing factors and the scope of community pharmacy practice in the care of clients with established CVD.

**Methods:**

In-depth, semi-structured interviews were conducted with 21 community pharmacists in New South Wales, Australia. All interviews were audio-recorded, transcribed *ad verbatim*, and analysed using a “grounded-theory” approach.

**Results:**

Our model shows that community pharmacists work within a complex system and their practice is influenced by interactions between three main domains: the “people” factors, including their own attitudes and beliefs as well as those of clients and doctors; the “environment” within and beyond the control of community pharmacy; and outcomes of their professional care. Despite the complexity of factors and interactions, our findings shed some light on the interrelationships between these various influences. The overarching obstacle to maximizing the community pharmacists’ contribution is the lack of integration within health systems. However, achieving better integration of community pharmacists in primary care is a challenge since the systems of remuneration for healthcare professional services do not currently support this integration.

**Conclusion:**

Tackling chronic diseases such as CVD requires mobilization of all sources of support in the community through innovative policies which facilitate inter-professional collaboration and team care to achieve the best possible healthcare outcomes for society.

## Introduction

Cardiovascular disease (CVD) is the leading cause of death in high- and middle-income countries, and ranks among the top five causes in low-income countries [1]. Guidelines are available covering recommendations about medical and lifestyle management for both primary and secondary prevention of CVD [2]. However, overcoming the negative attitudes of patients towards long-term preventative therapy is a challenge in the prevention of CVD [3]. Moreover, the use of secondary prevention medicines for CVD in high-, middle-, and low-income countries has been found to be suboptimal [4], while the provision of continuing support regarding lifestyle modifications to patients with established CVD after discharge following hospitalization has also been found to be lacking [5].

To strengthen the prevention of chronic diseases such as CVD, the mobilization of financial, human and technical resources through a cooperative and inclusive approach has been proposed [6]. This represents a momentum to enhance roles of untapped healthcare resources, including pharmacists [7].

Although the pharmacist was historically regarded as an “*assistant of the doctor*” and was responsible for preparation of medicines, since the birth of clinical pharmacy practice in the 1960 s, pharmacy has gradually evolved as a more clinically-oriented profession [8]. This prompted Hepler and Strand to coin the term “*pharmaceutical care*” to represent a philosophical change in professional pharmacy practice away from the dispensing role to a patient-centred role [9]. Importantly, they advocated for pharmacists to accept their “*social responsibility*” to ensure the safe and effective use of medicines. Moreover, they postulated that “*the fundamental goals, processes, and relationships of pharmaceutical care exist regardless of practice setting*” [9].

Pharmacists working in the community setting have traditionally been the first point of contact for medicine and health-related advice and are accessible without an appointment. According to the Australian Bureau of Statistics Census of Population and Housing 2011, approximately 80% of all employed pharmacists were community (or retail) pharmacists [10]. The supply of medicines, which is largely funded through the Australian Government’s Pharmaceutical Benefits Scheme (PBS), is still the main focus of the current community pharmacy business model. However, to engage pharmacists in helping to address gaps in healthcare especially focusing on safe and effective use of medicines, the Pharmacy Guild of Australia (the Guild, an employers’ organization servicing the needs of community pharmacists) strongly advocates an integrated, collaborative and sustainable business model through implementation of a number of professional health services by community pharmacists [11]. Under the 5^th^ Community Pharmacy Agreement (a negotiation between the Guild and the Government), professional services such as Home Medicines Reviews (HMRs) and Medicines Use Reviews (MedsCheck) are funded by the Australian Government [12]. To enable the delivery of such services, the Guild also highlights the need for community pharmacists to expand the scope of their role [11].

Using CVD as a case study, our recent publication reported a five-level model of scope of community pharmacist practice in the care of clients with established CVD, with the highest level of care consisting of comprehensive support on a continuing basis, covering counselling and monitoring medicines use, disease control, and lifestyle changes [13]. The practice of the majority of respondents, however, corresponded to the lower levels of the model since they only provided support on selected aspects of CVD management with some operating at the most basic level focusing only on medicines counselling. The findings, therefore, reflect the challenges faced by community pharmacists in implementing the pharmaceutical care model proposed by Hepler and Strand [9]. This paper focuses on an in-depth exploration of factors influencing community pharmacists in their everyday practice in the secondary prevention of CVD. Subsequently, we developed a model to explain relationships between these influencing factors and the scope of community pharmacy practice in the care of clients with established CVD published earlier [10].

## Methods

As previously published [13], an exploratory qualitative study using in-depth, semi-structured interviews was conducted using a ‘grounded-theory’ approach.

### Sampling and recruitment

The study was conducted in New South Wales (NSW), a state where the highest proportion (30%) of all employed pharmacists in Australia was located [10].

To enable sampling a wide variety of NSW community pharmacists working in different types of pharmacies, either independent or banner group pharmacy (groups that provide their members with common advertising and promotional support, advice on store layout and business practices [14]) in metropolitan and rural areas, a maximum variation sampling approach was applied. A list of community pharmacies was obtained from the Health Professionals Councils Authority – Pharmacy Council of NSW. As representation of accredited pharmacists to conduct HMRs was also required, a list of accredited HMR pharmacists in NSW was obtained from the Australian Association of Consultant Pharmacy website.

Invitation letters were sent through electronic mail or facsimiles to purposively selected pharmacists who were considered to meet the criteria. Pharmacists who had not responded to the invitation were contacted over the telephone. Subsequently, those who expressed interest to take part in the study were sent a participant information sheet. Once a pharmacist had agreed, a date, time and venue for an interview were arranged at their convenience. Face-to-face interviews were conducted with metropolitan pharmacists. Due to geographical restrictions, rural pharmacists were interviewed over the telephone or via Skype. Written consent was obtained from participants in face-to-face interviews, while verbal consent was obtained from interviews over the telephone or via Skype and documented in audio-tape recordings.

### Ethics statement

Prior to commencement of the study (May – November 2012), approval was obtained from the University of Sydney Human Research Ethics Committee (Ref No. 2012/407). This included approval for the method of obtaining either written or verbal consent from the participants.

### Data collection and analysis

An interview protocol was used to maintain consistency during interviews. The interview protocol was developed from a literature review of published articles on community pharmacist interventions in the management of CVD [15]–[20] and discussions among the investigators, and then revised following pilot interviews with two community pharmacists. Each interview was started by asking pharmacists about their awareness of CVD secondary prevention and the nature of support provided to clients with established CVD. (The findings of these two themes have been published earlier [13]). Following this, participants were asked about facilitators and barriers to supporting CVD secondary prevention (Table 1). Interviews were conducted until reaching ‘saturation’, when no new themes emerged in three consecutive interviews.

**Table 1:** Interview protocol.

QUESTIONS	PROMPTS
**Facilitators**	
What do you think are the factors that help you engage in activities which support secondary prevention of CVD among your clients?	• Personal factors: perceived importance? Job satisfaction?
	• Client factors: motivation?
	• Other HCP factors: expectation?
**Barriers**	
What prevents you from getting more involved in supporting secondary prevention of CVD?	• Personal factors: knowledge? Interest? Skills?
	• Client factors: health beliefs? Lack of motivation? Language barrier?
If **NOT** offering support to clients with CVD, what makes it difficult for you?	• Other HCP factors: poor communication?
	• Organizational factors: time? Staff? Private counseling area? Financial support?

HCP = healthcare professional.

All interviews were audio-recorded and transcribed *ad verbatim*. In parallel with interviews, data were entered into NVivo 9.0 software, analysed using a constant comparison method, and coded using an open and selective coding method [21]. Following the coding the investigators verified themes to generate a theory.

## Results

**Figure 1:**
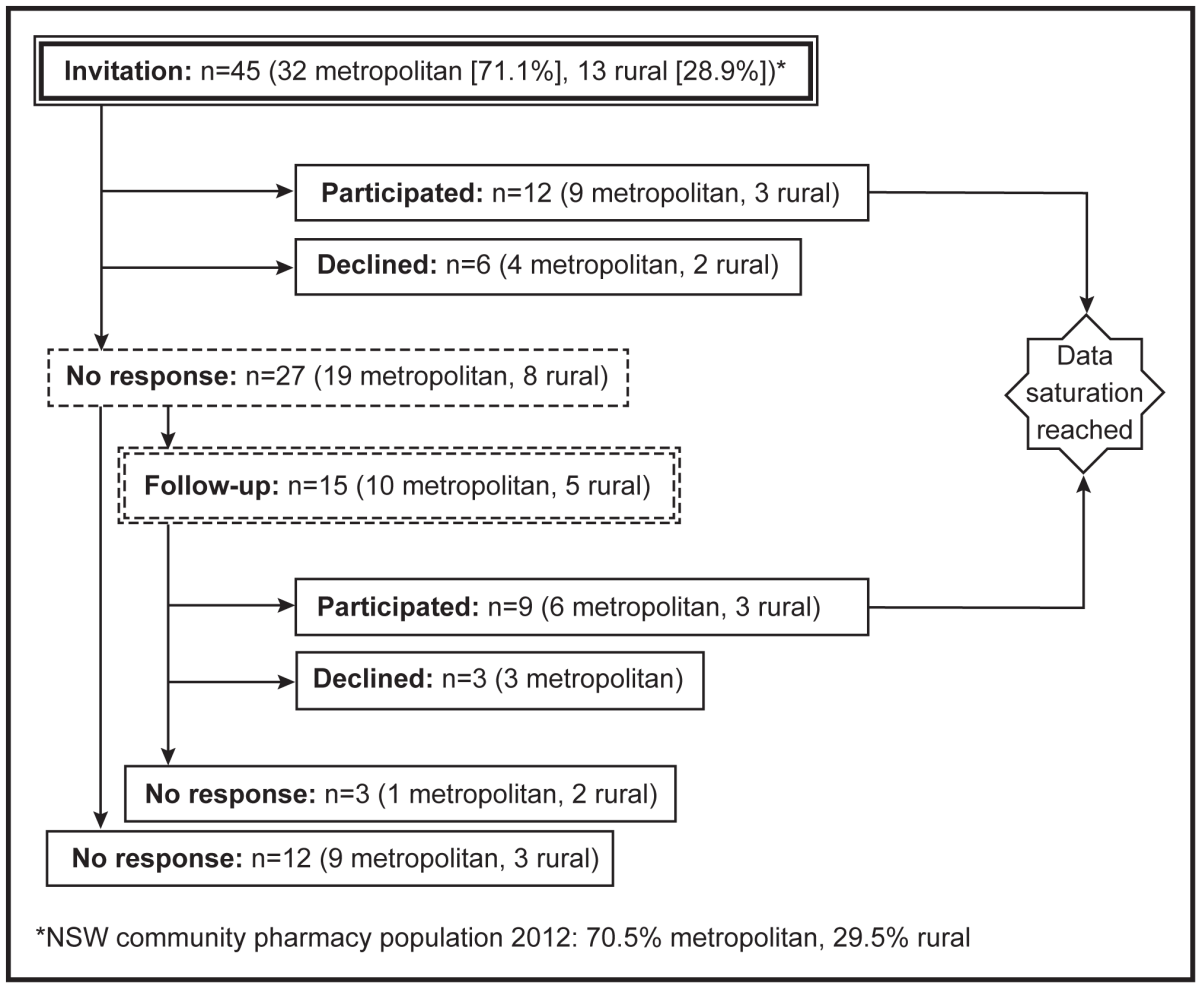
Recruitment process.

Forty five community pharmacists were invited to participate. Of these, 15 did not respond, nine declined and data saturation was reached after interviewing 21 pharmacists (Figure 1). The majority of participants were from metropolitan areas (71%) and worked in independent pharmacies (71%). Of 21 pharmacies, 76% employed more than one pharmacist on all business days and 52% dispensed more than 800 prescriptions per week. Most of the participants were female (67%), sole proprietor (43%), on average 45 years of age (range 24–66), with 21 years of experience as a community pharmacist (range 2–40) and 33 work hours per week (range 4–60). Nine participants were accredited Home Medicines Review pharmacists.

Three key themes identified as influences on community pharmacist practice in CVD secondary prevention were broadly classified as: ‘internal’, ‘external’ and ‘outcome’ factors. The data were further synthesized to propose a model to illustrate relationships among factors. Each theme is outlined below.

### Internal factors

Factors that influenced the pharmacist’s personal ability to provide support were categorized into ‘internal’ factors. Three sub themes emerged during data analysis, including attitude, knowledge, and communication skills, with quotes shown in Table 2.

**Table 2:** Quotes illustrating internal factors.

**Pharmacist attitude**
“I really don’t think that the doctors have time to sit with someone and go through the entire diet, to go through the actually doing exercise, you know.” … “I think there is a huge potential for pharmacists to be involved.” [*Rural*01-*female*]
“I see the doctor as the ultimate guru and really don’t want to intervene too much.” … “I don’t want to get involved in doctor’s therapy, not for heart disease, if they’ve established heart disease.” … “I don’t think it’s a big role of the pharmacists.” [*Metro*04-*female*]
**Pharmacist knowledge**
“There is so much you can do as a pharmacist. You try to educate people. You try to teach them how to take their medication regularly… then how to maintain a healthy lifestyle.” … “And I think that’s where the pharmacist’s role in preventative measures, it’s really important because we do have the knowledge.” [*Metro*15-*female*]
“But, we can’t start looking about heart disease specifically because we’re not really trained in that disease… We’re trained in medications. We’re trained to explain and how to dispense properly… Heart disease is very complicated and dangerous… I don’t think at the moment with the way we are as a profession should go any further from that (counselling on medications)… If in the future we do have specialized pharmacists and they’re trained in heart disease that’s fine.” [*Metro*04-*female*]
**Pharmacist communication skills**
“From my experience, if patients are really… appreciate the healthcare provider who provides care for them on, who I prepare to listen to their needs and wants and preferences… somebody [who] can spend time with them, … have an informal chat about the progress in a non-judgmental way, in a friendly and sort of collaborative environment.” [*Metro*12-*male*]
“But it’s probably challenging for me, for my age as well, because I think younger pharmacists coming out trained really well in that… interaction with doctors. You know, I’m quite aged. So in my training we didn’t do any of that when we were trained.” [*Metro*02-*female*]

#### Pharmacist attitude

Many respondents believed that community pharmacists have an important role to play in CVD secondary prevention. Elements of this role within their scope of professional practice included counselling the client not only about medicines but also their condition(s) and lifestyle as well as monitoring for medicine-related problems including adherence, lifestyle modifications, and disease or risk factor control. This was especially the case because it was believed that many doctors do not devote enough time to addressing the array of issues in CVD secondary prevention with clients. However, opposing views were expressed by several respondents who considered the doctor as the most appropriate healthcare professional to manage CVD.

#### Pharmacist knowledge

To be able to support clients with CVD, knowledge about CVD secondary prevention was considered important. The majority perceived that the pharmacist has sufficient knowledge of CVD and its management to offer support to clients with CVD. In contrast, others felt that they lacked knowledge about CVD and perceived specialization as a way of facilitating their support for clients with CVD.

The concept of specialization and accreditation for pharmacists involved in CVD secondary prevention was notably supported by respondents who were offering a higher level of care [13]). However, the majority did not favour specialization because this might limit their ability to provide support to clients with other disease states. The purpose of additional training was, therefore, to improve their knowledge.

#### Pharmacist communication skills

Many recognized that good communication skills are pivotal to providing support in CVD secondary prevention. Several respondents reported that by applying effective communication skills they were able to elicit information from their clients regarding their condition and how they managed their treatment regimens. As a result, the pharmacist could tailor appropriate recommendations to clients. Effective communication was also critical to creating a friendly and informal environment in which to foster and maintain long-term relationships with the client. Communication skills were also regarded essential to maintaining relationships with the doctor. The few who perceived that they lacked the skills were less likely to interact with the doctor.

### External factors

Other factors perceived by respondents to influence their activities in supporting clients with CVD were classified as ‘external’ factors. These factors related to the client, the doctor and other healthcare professionals, pharmacy environment, professional organizations and the health system/government policy, with quotes shown in Table 3.

**Table 3:** Quotes illustrating external factors.

**The client**
“I mean the obvious one is time and money… But, within that, there is obviously patient’s demand ‘cos not every patient wants it. So, you could say if you were given lots of money and you had the time, just spend half an hour for every patient or 20 minutes with the patient, they might not always want to.” [*Metro*11-*male*]
“I think they (the client) feel it’s the role of the GP to review them, to make sure that’s all good. And it’s our role to make sure that everything is ok with their medications. So, people have a perception in mind about what the roles of professionals are.” [*Metro*05-*female*]
“A lot of people go to a big medical centre and they don’t often… the doctors are not there every day.” … “So many things get missed because everybody (healthcare professionals) is busy. Everybody (clients) is running around [to different pharmacies]. And that’s why people should also stick to one pharmacy.” [*Metro*15-*female*]
**The doctor and other health care professionals**
“The barriers are strong in that… doctors don’t like you’re doing a lot of things. Oh yes, depending on the age of the doctor.” … “There are some people (doctors) that have no idea what I do for people (clients)… Because they feel threatened their work would be reduced. Absolutely. I’m sure of it.” [*Metro*15-*female*]
“It’s about good communication, not just writing a prescription. I mean, writing a legal and valid prescription is always wonderful, but also letting pharmacists to know that, ‘This is an increasing dose from…’ you know.” [*Rural*01-*female*]
“We don’t get it (response regarding pharmacist HMR recommendations) back from the doctor. Yeah. So, that’s an important communication tool… So ideally, that piece of document is the agreed plan and should be shared and communicated to the pharmacist who is going to follow up with the patient. I would say 90% of the time I don’t get that document, so it’s upsetting.” [*Metro*12-*male*]
“They (other healthcare professionals) are REALLY good.” … “[We refer the patient to] people that we have a formal relationship with already… [We] send the patient down with a note and the information on it. But if that’s for someone [who is] further away, then usually just the verbal thing that we say [to the patient].” [*Metro*06-*female*]
**Pharmacy environment**
“It depends on which pharmacy you dispense your prescription. If you dispense your prescription in a discount pharmacy where there are so many customers, and you only have two pharmacists on duty who are dispensing and handing medication out. Definitely, you would NEVER be able to speak and talk about the medication… just give it and hand it out, especially with your repeats.” [*Metro*14-*female*]
“I think you have to have a place in the pharmacy to roll out the service…because ultimately these people are gonna be with you for half an hour or a lot more. So, I think it’s about allocating a space in the pharmacy and making it a sustainable service.” [*Metro*05-*female*]
“We are quite lucky because we are the only pharmacy here on this street. I mean, we’re very different from [a] pharmacy around the corner. So it’s not like competition with each other. He is not a discount pharmacy. He is also professional advice… I mean, if we got a discount pharmacy in the area, I don’t know. That could change.” [*Metro*10-*female*]
“When you’re in this business and they’re expensive, you’re gonna think about money. It is pharmacy, it is really retail, it is 90% retail, you know, and 10% professional. That’s not a bad thing, that’s reality.” … “And so, we’re not gonna be sitting and writing notes because it takes time. And also, what benefit does it? The benefit is only minimal to the business. [The benefits] might be maximal for the person (client) but minimal [for business].” [*Metro*04-*female*]
**Professional organizations**
“I think the Guild and PSA (Pharmaceutical Society of Australia) should have played their role in enabling the pharmacist to better support patients. Like, for example we don’t even have guidelines for lifestyle management specific for the pharmacist.” … “And also documenting patients in pharmacy software is quite important. I think pharmacy… the design of the computer systems for pharmacy dispensing software does not support you to be able to assess patients, follow up the treatments, record important data about them.” [*Metro*12-*male*]
**Government policy**
“We do a lot on the spot. But the thing is being paid for that sort of thing.” … “Because I can see if the government continues down the track reducing the margin on drugs, something you’re not getting paid your… mark up to justify pharmacists to do intervention, then you’re just dispensing.” [*Metro*09-*female*]
“The most important thing that I found is high discharged from hospital… that’s an area that typically people get confused what medications are on, like prior to an event and after an event… So, I think moving off information is probably an important thing in continuity of care. REALLY REALLY important.” [*Rural*01-*female*]

#### The client

Lack of client interest or motivation to engage with any additional support offered by the pharmacist was identified by many as a significant barrier to an enhanced pharmacist role in secondary prevention of CVD. For example clients who were perceived to be highly educated and who believed that they were knowledgeable about their condition were often believed to be unreceptive to pharmacists’ offers of counselling. This discouraged pharmacists from pursuing the discussion. Another group of clients who were difficult to engage with were clients with language barriers and low health literacy.

Several respondents also indicated there was a view among clients that pharmacists had no role to play in the monitoring of their clinical conditions. These clients believed that it was solely the role of their doctor to provide such support. In turn, the client refused the offer of any monitoring by the pharmacist.

In addition, as providing support to clients with chronic disease such as CVD requires continuity of care, it was difficult to offer such care to non-regular clients, as well as those who do not see the same doctor for regular follow-up.

#### The doctor and other healthcare professionals

The majority acknowledged that a professional working relationship with the doctor was required to support clients with CVD. In this context, many perceived barriers were related to doctors’ attitudes and beliefs. There was a perceived lack of recognition by many doctors that pharmacists could contribute to secondary prevention and continuity of care in CVD. As a result, the doctor was perceived to be unreceptive to any pharmacist initiatives in engaging in collaboration. Difficulty in communication with some doctors also occurred in the context of interactions about prescription medicines.

In terms of HMR services, although a pharmacist-doctor collaboration is an integral part of the service, several accredited HMR pharmacists in this study reported that discussions with doctors regarding their HMR recommendations did not always occur. In order to develop a good working relationship with the doctor, many respondents expressed the need for delineation of roles of the pharmacist and the doctor.

In addition to working with the doctor, several participants mentioned that they worked with other healthcare professionals to support clients with CVD. Dieticians and physiotherapists were frequently mentioned, followed by diabetes educators, community nurses, and occupational therapists. Despite that, they acknowledged that their communication was primarily dependent upon their proximity and close relationships with these professionals.

#### Pharmacy environment

Several respondents reported that the support they offered to clients with CVD was constrained by time pressures related to workload and lack of staff. Some pharmacists mentioned that enhanced services were more likely to occur in community pharmacies when there were enough pharmacists so that some could look after routine such as dispensing, freeing up others to counsel clients. Moreover, more than half the participants acknowledged the availability of a private counselling area was another important element in offering such support.

Furthermore, many emphasized that it was challenging to offer support to clients with a chronic disease when they had restricted access to clients’ data. Having good documentation systems at pharmacy were also regarded crucial to help sustain their services. Of course, with non-regular clients documenting progress was difficult, precluding continuity of support. In this context, concern was expressed by several respondents about the trend to ‘discounting pharmacies’ that might hinder their ability to maintain their regular clients. Price competition among community pharmacies was driving clients to collect prescription medicines at discount pharmacies. In addition, it was acknowledged that implementing pharmacy advanced services that involve clinical interventions was challenging when a business orientation dominated.

#### Professional organizations

Although there was some appreciation for the remunerated professional pharmacy programs now available for community pharmacists in Australia, such as HMR and MedsCheck services [22], [23], some believed that professional organizations should offer more in terms of guidelines. The need for improvement in pharmacy computer systems was also noted.

#### Government policy

The main concern related to support from the government raised by many respondents was remuneration for providing services. It was also noted that the structure of health system needs to be improved, with regard to the accessibility of clients’ data.

### Outcome factors

As expected, a key motivation for providing services to clients with CVD was to help the client achieve better health outcomes. For many respondents, their motivation was reinforced when observing that the support they offered translated into tangible improvements in patient health. Finally, they acknowledged that not only does providing such support bring them job satisfaction, it also results in financial benefits for their pharmacy through client loyalty:

“They’re really like ethical and professional ones for me such, you know, you feel much more rewarded and satisfied with your work when you see patients are getting better more than… or the patient complimenting you for your help. So, that’s like a motivational aspect. I guess another thing would be… well you can’t ignore the financial side of things and of course that the patient is likely to be… to commit to your pharmacy and be a loyal customer, if you like. So, that’s of course I appreciate that. Of course it means that it’s better for your business as well.” [*Metro*12-*male*]

### A model of the determinants of pharmacists’ provision of support for CVD secondary prevention

**Figure 2:**
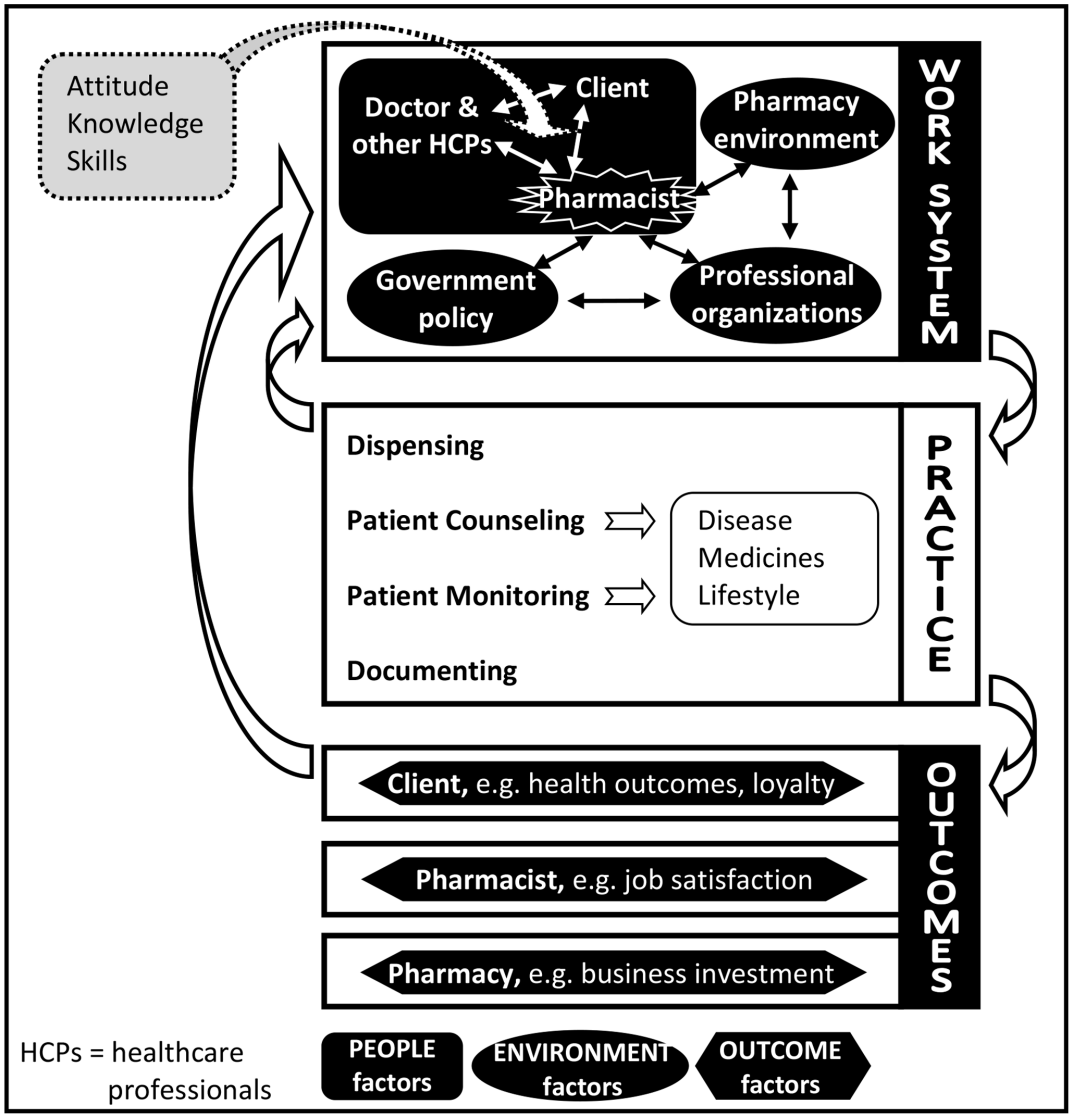
Relationships among factors influencing community pharmacist practice in supporting CVD secondary prevention.

Figure 2 illustrates relationships among factors influencing community pharmacist practice in providing support to clients with CVD. ‘Internal’ factors relating to the pharmacist together with factors related to the client and other healthcare professionals as described above have been re-grouped under ‘people’ factors in Figure 2 and they were identified as influenced by attitudes and beliefs. In the discussion, we describe the circle of ‘people’ as “*health team*”. The remaining ‘external’ factors have been renamed as ‘environment’ factors in Figure 2. Both ‘people’ and ‘environment’ factors, subsequently, are classified into ‘work system’ (Figure 2, top). Activities (details reported elsewhere [13]) conducted by community pharmacists to support clients with CVD including dispensing, patient counselling, patient monitoring, and documenting are grouped under ‘practice’ (Figure 2, middle). Finally, outcomes of pharmacist support to the client, the pharmacist or pharmacy are grouped together under ‘outcome’ factors (Figure 2, bottom).

## Discussion

The findings of this study highlight the challenges faced by community pharmacists in the implementation of pharmaceutical care for the secondary prevention of CVD. As primary healthcare professionals, they work within a complex system wherein their practice is influenced by factors within three main domains: the ‘people’ factors, including their own attitudes and beliefs as well as those of clients and doctors regarding their professional scope of practice; the ‘environment’ within and beyond the control of community pharmacy; and outcomes for their clients with CVD resulting from their professional care. In this article, we propose a model illustrating relationships among factors and also suggest that the circle of ‘people’ represents “*health team*”. To our knowledge, this model is the first holistic analysis of activities and elements that need to be considered when implementing pharmaceutical care in the community setting, using cardiovascular disease as a case study.

### The work system

#### Stakeholder attitudes – pharmacists, doctors, and patients

As in this study, other previous studies identified community pharmacist positive attitudes as a facilitator to provision of extended services beyond dispensing role [24], [25]. However, using the Pharmacy Asthma Management Service as a case example, Feletto *et al*. reported that even if pharmacists held positive attitudes and had the knowledge and competence to perform the service, they found actual service implementation was often difficult [25]. According to the literature, although the scope of pharmacy practice has evolved, doctors have continued to resist the extended role of pharmacists [26]–[28], while patients have been reluctant to accept that the pharmacist has a role in recommending treatments and continue to view the doctor as the most appropriate professional to provide this [29], [30]. Many pharmacists in our study also perceived barriers related to negative attitudes of doctors and clients towards pharmacist services in CVD secondary prevention. This accords with findings of a Canadian study, which identified that pharmacists considered limited relationships with doctors and patients as their greatest challenge in implementing practice change in the community setting [31]. These findings suggest that community pharmacists might find it hard to fulfil one of four criteria “*the provider must be able to develop the relationships with the patient and other healthcare professionals*” that must be met before providing pharmaceutical care [9].

In the Total Pharmacy Care Model, Holland and Nimmo defined pharmacist-patient-other healthcare professional relationships in pharmaceutical care practice model as “*patient care team*” [32]. Numerous trials designed to examine pharmacist contributions within a patient care team –mostly in the form of doctor-pharmacist teams– showed positive impacts on achieving patient outcomes [33]. However, the majority of trials were conducted within a controlled research environment, such that team-based models may not be easily translated into daily practice. For example, in a study which did not find significant impacts, there were difficulties faced by community pharmacists in contacting patients or caregivers and scheduling appointments and this hindered the implementation of pharmaceutical care [34]. As the pharmacists in our study, who mostly deal with regular clients, were more likely to offer a higher quality of care [13], we propose in our model that the pharmacy client needs to be considered as an integral part of “*health team*”.

A view to ideally include the patient as an active member of primary care teams has also been suggested previously [35]. In addition, it has been reported that ‘active’ patients can be the main driver for developing inter-professional collaborations [36]. Based on our findings, the likely approach to integrating clients in the “*health team*” in the community setting is by advising clients to visit a regular pharmacy. However, it is obviously difficult as clients have the right to select services, especially when they are offered at competitive prices [37].

To allow healthcare professionals to work in a team, the benefit of co-location has been discussed and considered as a key to successful integration [36] because “*the fact that individuals and organizations are interconnected, …[provides]… places for discussion and for constructing bonds between them*” [38]. However, co-location does not necessarily guarantee that team members work together when little interaction occurs and time constraints for face-to-face communication persist [36]. For example, ineffective communication among healthcare professionals due to poor coordination and disorganisation has been reported as a barrier to optimal continuity of care for patients with heart failure [39]. Therefore, it is clear that coordination through structured programs is needed to optimize the care of patients with chronic diseases in the community setting. This issue is further discussed below under sub-heading ‘government policy’.

#### Knowledge and skills

Although lack of clinical knowledge was reported a barrier, in our study, the issue was raised by both pharmacists who felt that they lacked knowledge about CVD and those who already offered a higher level of care [13]. The latter cohort expressed the need for pharmacists to obtain accreditation to conduct advanced clinical services. In the Australian context, this refers to HMR services that can only be conducted by registered pharmacists who have completed the appropriate level of training and credentialing [22], in addition to their pharmacy training through a Bachelor of Pharmacy or a graduate entry Master of Pharmacy, followed by an intern training program that comprises 1,824 hours of supervised practice [10].

Furthermore, negative attitudes towards an extended pharmacist role beyond dispensing activity expressed by some participants in our study may reflect their lack of knowledge about the changing landscape of healthcare in Australia. A previous Australian study also reported low awareness of Australia’s healthcare reform agenda among community, hospital and consultant pharmacists, and their beliefs and attitudes toward supervision and supply of medicines as their role in the healthcare system [40]. Mak et al. suggested strategies to raise awareness within the profession through providing information and education as well as applying appropriate role models and practice sites during pre-registration pharmacy education are required to facilitate the adoption of the pharmaceutical care model [40].

Lack of knowledge and recognition from clients and doctors about the expanding role of pharmacists was also indicated by some participants in our study as a barrier to communication with the two cohorts. Indeed, this represents a considerable challenge in implementing the concept of a “*health team*”. Understanding each team member is a key in teamwork [41] and it is important that all members of “*health team*” (referring to our model) not only need to adopt positive attitudes, but also recognize each other’s roles and have ability to communicate with each other. As has been noted by the Guild that the community pharmacy network is still “*under-utilised by governments at all levels and also by other elements of the health system*” [11]. The pharmacy profession needs to be clearly identified in national healthcare reform documents to help improve clients’ and doctors’ understanding of pharmacists’ enhanced role.

#### Pharmacy environment

As with our findings, other studies reported that implementation of extended community pharmacist services has been influenced by factors within community pharmacy, such as the availability of private counselling areas, documentation systems and the number of pharmacists working [24], [42]–[46]. Some participants in our study also felt that the price competition among community pharmacies due to the increasing trend of discount pharmacy is likely to reduce their ability to sustain clinical interventions to their clients. Therefore, pharmacy owners/managers need to take responsibility for creating a practice environment that enables their pharmacists to provide high quality of patient care. Moreover, leadership from community pharmacy owners/managers is required to motivate pharmacist staff to accept practice change [24], [45], [46].

#### Professional organizations

Leadership internal to the pharmacy alone will not suffice. Leadership from pharmacy organizations is essential for implementation of advanced services [24], [46]. Pharmacists in our study expected support from professional organizations in the forms of practice guidelines and improvements in pharmacy computer systems. Furthermore, as shown in our model, professional organizations play a role in bridging the gap between pharmacist practitioners, policy makers and other key stakeholders [11].

#### Government policy

With regards to HMR services, we found that this government funded program allows community pharmacists to demonstrate comprehensive patient care practice [13]. There are reasonable explanations for this. Firstly, the application of a structured program enables community pharmacists to build relationships with their clients as well as professional collaboration with doctors and other healthcare professionals [22]. Looking at our model, this could drive the circle of “*health team*” within the work system. Such collaboration could develop trust, which might then lead to a change in clients’ and doctors’ attitudes towards pharmacist advanced services. Secondly, such a structured program also allows pharmacists to access their clients’ clinical data required in conducting clinical assessment and recommendations [22].

The availability of remuneration, which was of great concern to many respondents, could also motivate pharmacists to implement advanced services. Some researchers categorized remuneration as an external factor related to the government [25], [46], while other studies classified financial support as an internal factor to the pharmacy [24], [43]–[45]. However, many authors concluded that challenges for pharmacists in providing advanced services under team-based care models include lack of payment mechanisms and recognition as providers in government structured programs [7], [47]. White added that although drug costs may increase as a result of pharmacist interventions, “*it is not realistic to fund these activities as a pharmacy benefit… should be paid for as part of the medical benefit*” [7]. Therefore, changes in a national healthcare system are critical to enable a country to adopt an expanded role for community pharmacists [48].

### Outcomes of pharmacist support

Consistent with our findings, pharmacists in other studies recognized that their involvement in enhanced professional services increases their awareness to be more ‘proactive’ to help patients achieve health outcomes, increases patient loyalty and creates demand within their existing market [25], [45]. Moreover, being proactive may be taken as a key to lead practice change within pharmacy organizations [25]. In addition, as effectiveness of an organization depends on its ability to satisfy the needs and demands of employees, owners, and clients [49], our findings reflect that outcomes of pharmacist support can be valued as drivers for implementation of pharmaceutical care.

### Social, political and economical implications

According to management theory [49], a small organization is generally vulnerable to “*task environment*”, referring to factors external to the organization. In the context of community pharmacy, based on findings of this study and other studies [24], [25], [42], [43], [46], it is clear that these external factors include interactions with clients, professional relationships with other healthcare providers, leadership and practice standards from pharmacy organizations, and support from healthcare authorities or the government. It is understandable because community pharmacists are part of complex social fields with various role expectations, from patient care to strong business performance [50], [51] and these challenge community pharmacists to implement practice change. Furthermore, looking at policy and legislative viewpoints, there are conflicts of interest to integrate lessons learned from research into practice [52]. Kerner also highlighted the challenge in building bridges between research and practice if there is lack of coordination between government agencies and researchers [52].

Despite multifaceted factors, our findings shed some light on collaboration across components and organizations, as suggested previously [53]. In this context, community pharmacists need to be better integrated within the health systems. To apply such a policy, some rules such as “*citizens are entitled to basic health services*” and “*providers are to be reimbursed for value*” should be widely accepted [53]. Finally, although national politics and economy largely shape the future of health-system pharmacy in a country [54], we suggest that such government policies with potential to bring social benefits should not be put aside, but seriously considered.

## Limitations

Due to the nature of the study, the findings may not necessarily be generalized to the whole Australian pharmacist population. However, the findings have been generated from a wide variety of community pharmacists, with respect to gender, position in community pharmacy, years of practice experience, who worked in different types of pharmacies and locations (metropolitan and rural areas), which are likely to represent Australian community pharmacists in general. Moreover, generalization of the findings to community pharmacists globally may be inappropriate due to different health system among countries [55].

## Conclusion

In the provision of support to clients with established CVD, community pharmacists were not only influenced by their ‘internal’ factors and other factors related to people surrounding them, such as clients, doctors and other healthcare professionals, but also pharmacy environment, professional organizations and government policies. These factors appeared to correlate with one another to create a work system for community pharmacists. Within this complex work system, the attitudes and beliefs of pharmacists’ themselves as well as those of clients’ and doctors’ regarding the pharmacists’ professional scope of practice appear to be key determinants of actual practice behavior. Additionally, from the pharmacists’ perspective, ‘outcome’ factors generated their motivation for practice.

To enable targeting strategies to strengthen community pharmacists in implementing the philosophy of pharmaceutical care, further research is needed to examine predictors of actual practice in the care of clients with established CVD.
